# Transgene-free Genome Editing in Plants

**DOI:** 10.3389/fgeed.2021.805317

**Published:** 2021-12-02

**Authors:** Xiaoyong Gu, Lijing Liu, Huawei Zhang

**Affiliations:** ^1^ The Key Laboratory of Plant Development and Environmental Adaptation Biology, Ministry of Education, School of Life Sciences, Shandong University, Qingdao, China; ^2^ Institute of Advanced Agricultural Science, Peking University, Weifang, China

**Keywords:** genome editing, CRISPR/Cas9, transgene-free, transgene integration, editor delivery

## Abstract

Genome editing is widely used across plant species to generate and study the impact of functional mutations in crop improvement. However, transgene integration in plant genomes raises important legislative concerns regarding genetically modified organisms. Several strategies have been developed to remove or prevent the integration of gene editor constructs, which can be divided into three major categories: 1) elimination of transgenic sequences via genetic segregation; 2) transient editor expression from DNA vectors; and 3) DNA-independent editor delivery, including RNA or preassembled Cas9 protein-gRNA ribonucleoproteins (RNPs). Here, we summarize the main strategies employed to date and discuss the advantages and disadvantages of using these different tools. We hope that our work can provide important information concerning the value of alternative genome editing strategies to advance crop breeding.

## Introduction

Genome editing is a revolutionary technology for the advancement of plant science and crop breeding (Chen et al., 2019). The technique is based on site directed nucleases (SDNs), including meganucleases, Zinc-finger nucleases (ZFN), transcription activator like effector nucleases (TALEN) and clustered regularly interspaced short palindromic repeats (CRISPR)/CRISPR-associated (Cas) system (Gaj et al., 2013; Puchta and Fauser, 2014). Due to its simplicity and easy manipulation, the CRISPR/Cas system is ubiquitously used in the development of genome editing tools (Kantor et al., 2020). The basic CRISPR/Cas system requires two components: a Cas nuclease, such as Cas9, Cpf1 and a guide RNA (gRNA) (Zetsche et al., 2015; Jiang and Doudna, 2017). The gRNA can be programmed to bind to target DNA, and direct the Cas nuclease to perform a double-strand break (DSB) within the target site. DSB repair in plants is majorly achieved through an error prone non-homologous end joining (NHEJ) pathway, which usually leads to some base insertions/deletions (indels) and generates mutations at the target site (Jiang and Doudna, 2017). To date, several base and prime editor tools were developed based on CRISPR/Cas in order to perform more precise editing (Zhu et al., 2020). These editing tools are helping breeders modifying target genes to the desired sequence for improving crop yield and quality, and increase biotic/abiotic stress tolerance and herbicide resistance in crops (Chen et al., 2019). Genome editing is therefore considered designated the next generation breeding strategy.

Legislation and regulation are critical for marketing approval of edited crops (Jones, 2015). Genome editing generates small indels, base-pair changes and specific short sequence changes through HDR (homologous recombination) that are indistinguishable from natural genome variants. Accordingly, in several countries and geographical regions, these types of mutants are not categorized genetically modified organisms (GMO), and are thus exempted from GMO regulation (Kim and Kim, 2016; Turnbull et al., 2021). Obviously, a major challenge for the application of genome editing in crop breeding is generating transgene-free edited plants.

**FIGURE 1:**
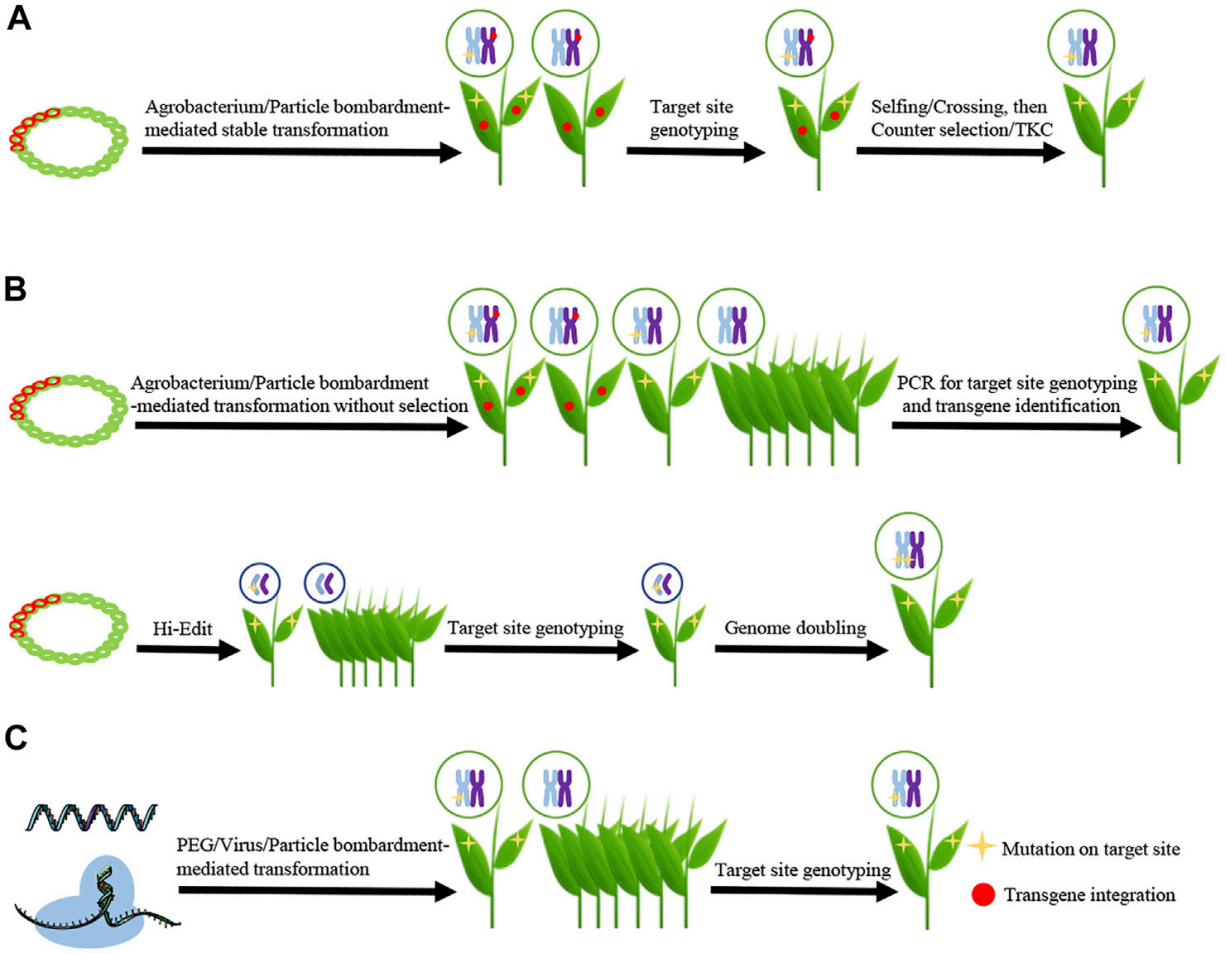
Schematics showing the main strategies for isolating transgene-free and genome-edited plants **(A)** Eliminating transgenic sequence through genetic segregation. CRISPR/Cas DNA (represented by red double helix) is delivered into plant cells using *Agrobacterium tumefaciens* or particle bombardment. The transgenic plants are isolated, and then genome edited plants are selected through target site genotyping. The transgene-free and genome edited plants are isolated from progenies of transgenic genome edited plants which is facilitated by counter-selection or transgene killer CRISPR (TKC) **(B)** Transiently expressing the editor from DNA vectors. CRISPR/Cas DNA could be delivered into plant cells using *Agrobacterium tumefaciens*/particle bombardment (upper schematic) or Hi-Edit (lower schematic). For the strategy using agrobacterium/particle bombardment-mediated transformation, transgene-free and genome edited plants are isolated from all the regenerated seedlings by PCR for target site genotyping and transgene identification. For the strategy using HI-Edit, the haploid progenies are selected and genotyped. The genome edited haploid plants are genome doubled to produce the transgene-free and genome edited doubled haploid line **(C)** Delivering editors in a DNA-independent manner. CRISPR/Cas9 RNA or Ribonucleoproteins (RNPs) are delivered into plant cells by polyethylene glycol (PEG)-, virus- or particle bombardment-mediated transformation, and then transgene-free and genome edited plants are isolated from all the regenerated seedlings by target site genotyping. Mutation on target site is represented by yellow star and transgene integration by red dot.

Conventionally, editor genes are placed in DNA constructs and then delivered to various plant cells using *Agrobacterium tumefaciens* or particle bombardment-mediated transformation (Altpeter et al., 2016). With selection markers, such as antibiotic or herbicide-resistant genes, the first generation (T0) transgenic plants are isolated, and genome edited plants distinguished from transgenic plants through DNA sequencing (Yin K. et al., 2017). In order to obtain transgene-free edited plants, it is necessary for the integrated foreign DNA to segregate out via selfing or crossing with wild-type plants (Gao, 2021). This is a labor intensive and time-consuming process, and thus not suitable for several plant species. Here, we summarize the current strategies used to remove or avoid the integration of foreign transgene DNA in edited plants (Figure 1), discuss the advantages and disadvantages of each strategy, and evaluate the forthcoming challenges for the widely application of these strategies in crop improvement.

### Eliminating Transgenic Sequences Through Genetic Segregation

Stable transformation-mediated genome editing is suitable for most plants. Despite being a very simple and efficient strategy, isolating second generation (T1) non-transgenic edited plants is labor and time intensive. Hence, a handful of methods were developed to facilitate this process (Figure 1A).

### Transgene Counter-selection Strategies

To select the transgene-free plants from the progenies of transgenic genome edited plants, some visible selection markers were introduced. In *Arabidopsis*, Gao et al. inserted an mCherry expressing cassette into the CRISPR/Cas9 construct, driven by the seed-specific promoter *At2S3* (Xi et al., 2010; Gao et al., 2016). The transgenic seeds exhibit red fluorescence and can be visually detected in T1. This fluorescence marker-assisted system saves time for plant growth, genomic DNA extraction and genotyping. This strategy is suitable for establishing high-throughput and automated sorting systems.

Lu et al. developed an RNA interference (RNAi)-based strategy, termed CRISPR-S, in order to select transgene-free progenies in rice (Lu et al., 2017). The rice P450 cytochrome protein CYP81A6 confers plant resistance to bentazon, a commonly used herbicide (Pan et al., 2006). The addition of a CYP81A6-hpRNAi expression cassette to a CRISPR/Cas9 construct resulted in bentazon-hypersensitive transgenic plant. After spraying 1,000 mg/L of bentazon to four-leaf-stage seedlings of T1 edited lines, the transgenic plants started dehydrating and/or died. In contrast, while transgene-free seedlings were able to grow normally (Lu et al., 2017). The authors demonstrated all bentazon-resistant plants are transgene-free in their report.

Stuttmann et al. employed three transgene counter-selection markers in tobacco (*Nicotiana. benthamiana*) (Stuttmann et al., 2021). The fluorescence-based marker used the promoter of oleosin-coding genes from tomato to drive the expression of the fluorescence protein. All plants grown from non-fluorescent seeds of genome edited plants were transgene-free. The remaining two transgene counter-selection markers used were pepper’s *Bs3* gene (*Ca-Bs3*), which operated under the control of its own promoter (Romer et al., 2007); and a fusion protein comprised by the yeast cytosine deaminase coding gene (*ScFCY*) and the *E. coli* phosphoribosyl transferase-coding gene (*EcUPP*), which was driven by the *Arabidopsis* ubiquitin promoter. The results showed that *Ca-Bs3* caused cell death when induced by the effector *AvrBs3* (Boch et al., 2014). Hence, transgene-free plants could be detected by infiltration with a *Pseudomonas fluorescens* strain expressing *AvrBs3*. FCY converts nontoxic 5-fluorocytosine (5-FC) into the toxic antipyrimidine 5-fluorouracil (5-FU), which blocks thymidine synthetic processes and is incorporated into DNA and RNA (Mullen et al., 1992; Longley et al., 2003). In addition, EcUPP enhances the toxicity of 5-FU (Tiraby et al., 1998). These results demonstrate that the use of this marker allows for an easy screening of transgene-free progenies by adding 5-FC into the medium.

### Transgene Killer CRISPR (TKC) System

He et al. developed an TKC system in rice to enable active and automatic self-elimination of the transgene in edited progenies (He et al., 2018; He et al., 2019). The TKC technology works by adding two cassettes expressing the suicide genes *barnase* and *CMS2* into the CRISPR/Cas9 construct. *Barnase* is a bacterial gene encoding for a toxic protein with nuclease activity that is able kill plant cells (Mariani et al., 1990). The expression of *barnase* is driven by the promoter of rice’s early embryo specific gene *REG2*, which ensures the gene is solely expressed during early embryonic stages (Sun et al., 1996). In addition, CMS2 specifically disrupts mitochondrial functions during the development of the male gametophyte and causes male sterility (Wang et al., 2006; Hu et al., 2012). The expression of this gene is under the control of the *35S* promoter. This strategy allows Cas9 to edit target genes during transformation, and during callus and vegetative growth stages of T0 plants. When T0 plants undergo reproductive growth, these suicide genes are either expressed (*barnase*) or begin to work (CMS2). The pollen and embryos containing the transgenes are therefore killed, which ensures that all harvested seeds are transgene-free. In total, 203 T1 plants were analyzed by He et al., none of which were transgenic (He et al., 2018).

### Transiently Expressing the Editor From DNA Vectors

Although eliminating transgenic sequences through genetic segregation has been successfully performed in the majority of edited plant species, this strategy needs sexual segregation and thus takes an extra generation to be effective. This makes it time consuming and not suitable for plants with long juvenile stages, such as pear, or vegetatively propagated plants, including potato and strawberry.

Transient expression of CRISPR/Cas9 DNA through particle bombardment-mediated transformation.

The ability of particle bombardment to mediate transient transgene expression is well-recognized (Takemoto and Jones, 2014; Ozyigit and Yucebilgili Kurtoglu, 2020). Hence, Zhang et al. developed a transiently expressed CRISPR/Cas9 DNA (TECCDNA)-based genome editing system to avoid transgene integration (Zhang et al., 2016). Specifically, using the TECCDNA system, the authors successfully introduced constructs expressing gRNA and Cas9 into immature wheat embryos using particle bombardment. After this, the seedlings were regenerated without any selection pressure and sequenced (Figure 1B). The frequency of mutagenesis, estimated by dividing the number of regenerated mutants by the total number embryos used in the bombardment experiment, was estimated between 2.6 and 5.0%. The frequency of transgene-free genome edited plants was determined by PCR and estimated between 43.8 and 86.8% of the T0 mutants (Zhang et al., 2016).

Transient expression of CRISPR/Cas9 DNA through A. tumefaciens-mediated transformation.

*A. tumefaciens* is also able to mediate transient transgene expression. Accordingly, Chen et al. established a method similar to TECCDNA in tobacco (Chen et al., 2018). Specifically, tobacco leaf-disc explants co-incubated for 3 days with *Agrobacterium* harboring the Cas9 and sgRNA *PHYTOENE desaturase* ***(*** *PDS)* construct were used for callus induction and seedling regeneration without any selection (Wang et al., 2009). Among the regenerated seedlings obtained from 415 explants, a total of 197 exhibited an albino phenotype with a mutagenesis frequency of 47.5% (calculated as the number of mutants over the total number of explants used for infection) or 2.57% (calculated as the number of mutants over the total number of regenerated seedlings) (Chen et al., 2018). Among all *pds* plants, 17.2% were transgene-free.

### Haploid Induction (HI) Editing Technology (Hi-Edit)

Since most crop varieties are recalcitrant to *A. tumefaciens* - and/or particle bombardment-mediated CRISPR/Cas9 delivery, Kelliher et al. established the Hi-Edit method to directly edit elite inbred lines by crossing in maize (Kelliher et al., 2019) (Figure 1B). In the Hi-Edit method, the CRISPR/Cas9 construct was firstly transformed to NP2222, which is a common line used for transformation. The Cas9^+^ progenies from regenerated plants were crossed with a native haploid-inducer line, RWKS, to select F2 individuals that are homozygote for both the haploid inducing gene and the Cas9 insertion. The pollens from these F2 individuals were used to fertilize the egg cells of the elite inbred lines. Finally, the transgene-free mutant of interest could be identified in the descendant haploid progenies. Genome editing was achieved in five out of six maize elite inbred lines with >3% editing ratio in haploid progenies (Kelliher et al., 2019). These mutants were transgene-free, since they lacked the Cas9-containing DNA from the haploid inducer parent. Hi-Edit can also be applied to dicotyledons, such as *Arabidopsis*.

### Delivering Editors in a DNA-INDEPENDENT Manner

Editors can be also delivered in a DNA-independent manner, including *in vitro* transcribed RNA or preassembled Cas9 protein-gRNA ribonucleoproteins (RNPs) (Figure 1C). Because no transgene is involved in this process, all edited plants are transgene free.

### Transient Expression of CRISPR/Cas9 RNA (TECCRNA)-Based Genome Editing Method

In the TECCDNA system, it is possible that some small degraded vector fragments are integrated into the plant genome and difficult to detect by PCR. In order to avoid this possibility, the TECCDNA method was optimized to the TECCRNA system (Zhang et al., 2016). In this improved method, RNA is used as a vector (instead of DNA) to deliver the Cas9/sgRNA editor. The *in vitro Cas9* and sgRNA transcripts were introduced in immature wheat embryos using particle bombardment, and the seedlings regenerated without any selection pressure. A 1.1% mutagenesis frequency was detected in the TECCRNA system (corresponding to 17 T0 mutants over 1,600 bombarded immature embryos) with *TaGW2* sgRNA (Yang et al., 2012). Among these, 35.3% (6/17) contained a mutation in all six *TaGW2* alleles (Zhang et al., 2016). Since RNA molecules are unlikely to integrate into the plant genome, all of the TECCRNA mutants should be transgene-free.

### RNA Virus-Mediated CRISPR/Cas9 Delivery

Engineered virus vectors are used in biomedicine to deliver the CRISPR/Cas9 reagents into human cells (Yin H. et al., 2017). In plants, the *sonchus yellow net rhabdovirus* (SYNV), which is a negative-stranded RNA virus, was used by Ma et al. to deliver the Cas9 and the sgRNA encoding RNA sequence into tobacco leaves (Wang et al., 2015; Ma et al., 2020). The Cas9 and sgRNA sequence were inserted into the SYNV genome and their expression driven by native viral promoters. Two pre-tRNA_Gly_ were applied to the flanking regions of the sgRNA sequence to ensure sgRNA activity (Xie et al., 2015). The engineered SYNV was transformed into agrobacteria and then infiltrated into tobacco leaves. Systemic leaves were analyzed for mutagenesis efficiency (instead of infiltrated leaves), which ranged from 40 to 91% (Ma et al., 2020). The systemic leaves were further used for plant regeneration without selection, with >90% of the regenerated plants harboring mutations on the target locus (57% of which were inheritable) (Ma et al., 2020). Importantly, the progenies of the regenerated mutants were all virus free.

### Preassembled CRISPR/Cas9 Ribonucleoproteins (RNPs)-Mediated Genome Editing

Ribonucleoproteins (RNPs) composed of Cas9 protein and *in vitro* transcribed sgRNA have also been delivered into diverse plant cells for transgene-free genome editing (Woo et al., 2015; Svitashev et al., 2016; Liang et al., 2017; Park and Choe, 2019). RNPs were successfully delivered into the protoplasts of tobacco, Arabidopsis, lettuce, and rice, as well as to rice zygotes using polyethylene glycol–calcium (PEG–Ca^2+^)-mediated transfection (Woo et al., 2015; Toda et al., 2019). RNPs were also introduced into embryonic maize and wheat cells by particle bombardment. After RNP induction, the plants were regenerated from these cells without any selection. The mutagenesis efficiency of RNPs varied considerably. For example, up to 46% of the induced lettuce calli from RNP-transfected protoplasts were mutated, and the mutation was transmitted to the progenies (Woo et al., 2015). The proportion of mutants ranged from 14 to 64% of the total regenerated rice plants from RNP-transfected zygotes, and 1.3–4.4% of RNPs delivered by particle bombardment in wheat (Liang et al., 2017; Toda et al., 2019). Because no foreign DNA was introduced during CRISPR/Cas9 RNP mediated genome editing, the mutants obtained were completely transgene-free.

## Discussion

CRISPR/Cas9 system-mediated genome editing leads to efficient target modification in plants, including the model plant Arabidopsis and several crop species (Chen et al., 2019; Kong et al., 2021). This technology thus promises to accelerate basic research and crop improvement. Importantly, the elimination of CRISPR/Cas9 integration is highly desirable for gene functional studies and public acceptance of genome edited crops. The several strategies designed to avoid transgene incorporation were summarized in this mini-review (Figure 1).

Plant genome editing generally relies on *Agrobacterium*- and/or particle bombardment-mediated delivery of DNA carrying CRISPR/Cas9 reagents (Altpeter et al., 2016). All seedlings should be transgenic if the plant regeneration procedure is achieved under selection, with transgene-free plants being screened out from their progenies. The transgene-counter selection and TKC strategies were developed to facilitate this process (Gao et al., 2016; Lu et al., 2017; He et al., 2018; Stuttmann et al., 2021). Plants can also be regenerated without selection, although the transgene-free edited plants are often detected with lower efficiency, as a significant number of unmutated plants also regenerate (Zhang et al., 2016; Chen et al., 2018). There is a need to overcome a variety of persisting problems to facilitate the future application of *Agrobacterium*- and particle bombardment-mediated DNA delivery of CRISPR/Cas9. For example, not all crop varieties can be transformed or regenerated after transformation (Anjanappa and Gruissem, 2021); part of the CRISPR/Cas9 construct might integrate into the plant genome, and avoid detection by PCR (Zhang et al., 2016); particle bombardment causes genomic damage (Ozyigit and Yucebilgili Kurtoglu, 2020); most importantly, the identification of transgene-free genome edited plants using *Agrobacterium*- and particle bombardment-mediated DNA delivery is laborious and time consuming, independently of whether selection was applied or not during the regeneration process (Zhang et al., 2016; Chen et al., 2018; He et al., 2018).

To completely avoid DNA integration, RNA and RNPs are used to express CRISPR/Cas9 reagents in plant cells (Zhang et al., 2016; Park and Choe, 2019). These methods also decrease the off-target mutations, which remains a major concern of CRISPR/Cas9 integration (Zhang et al., 2015; Zhang et al., 2016; Zhang et al., 2018), and thus have a good prospect of commercialization. However, the difficulty to deliver RNPs limits their readily implementation by most labs (Woo et al., 2015; Subburaj et al., 2016). In addition, it is necessary to solve problems associated with the use of different types of plant cells as the target of CRISPR/Cas9 expressed from *in vitro* transcribed RNA or RNPs. In cases where embryonic cells are used, the mutagenesis efficiency is relatively low since the vast majority of regenerated plants are unmutated (Liang et al., 2017). The mutagenesis efficiency increases when protoplasts are used (Woo et al., 2015). However, it remains technically challenging to isolate, culture and regenerate plants from protoplasts across several important crops (Lin et al., 2018). The use of RNA viruses to deliver CRISPR/Cas9 expressing RNA into plant cells, likely constitutes the most convenient and efficient strategy to generate transgene-free genome edited plants at present (Ma et al., 2020). However, constraints regarding the host range associated with specific viruses remains an important limiting factor to the implementation of this strategy (Dawson and Hilf, 1992). To date, RNA virus-mediated CRISPR/Cas9 delivery is only applicable in tobacco. We highlight the need for the development of new delivery strategies for CRISPR/Cas9 RNA and RNPs in order to improve delivery efficiency, and build more robust screening systems to distinguish transgene-free mutants from unmutated samples. These advances are urgently needed to promote the application of CRISPR/Cas9 technology in agriculture.
